# Comparison of the prognostic impact of IPI and PIT in peripheral T-cell lymphoma in real-world practice with a large elderly population

**DOI:** 10.1038/s41598-023-46501-5

**Published:** 2023-11-04

**Authors:** Nobuhiko Nakamura, Nobuhiro Kanemura, Takuro Matsumoto, Hiroshi Nakamura, Yoshikazu Ikoma, Yuhei Shibata, Junnichi Kitagawa, Senji Kasahara, Toshiki Yamada, Michio Sawada, Yuto Kaneda, Kenji Fukuno, Eri Takada, Hideko Goto, Shin Lee, Kei Fujita, Tetsuji Morishita, Takeshi Hara, Hisashi Tsurumi, Masahito Shimizu

**Affiliations:** 1 Department of Hematology and Infectious Disease, Gifu University Hospital, Gifu, Japan; 2 Department of Hematology, Gifu Municipal Hospital, Gifu, Japan; 3 Department of Hematology, Gifu Prefectural General Medical Center, Gifu, Japan; 4 Department of Hematology, Gifu Red Cross Hospital, Gifu, Japan; 5 Department of Hematology, Takayama Red Cross Hospital, Takayama, Japan; 6 Department of Hematology, Gihoku Kosei Hospital, Yamagata, Japan; 7 Department of Hematology, Chuno Kosei Hospital, Seki, Japan; 8 Department of Hematology, Matsunami General Hospital, Gifu, Japan

**Keywords:** T-cell lymphoma, T-cell lymphoma

## Abstract

We compared the predictive ability of the International Prognostic Index (IPI), a frequently used prognostic model for peripheral T-cell lymphoma (PTCL), with that of a type-specific prognostic model, the Prognostic Index for PTCL-U (PIT). We retrospectively analyzed 113 patients diagnosed with PTCL. The median age was 67 years (range, 16–88 years), 75 patients (66%) were male, and the most common disease type was PTCL, not otherwise specified (69%). With a median follow-up of 6.8 years (interquartile range, 2.7–9.9 years), 5-year survival rates for the four groups in IPI were 85%, 62%, 49%, and 13%, respectively. Similarly, 5-year survival rates for the four groups in PIT were 83%, 64%, 49%, and 19%, respectively. The area under the receiving operating characteristic curve for predicting mortality from PIT (0.725) was not significantly different from that from the IPI (0.685, *P* = 0.134). Multivariable analysis showed that performance status ≥ 2 (*P* < 0.0001) and extranodal lesions ≥ 2 (*P* = 0.029) were significantly associated with lower overall survival. The present study found no significant difference in prognostic ability between the IPI and PIT for PTCL, and both models appear useful as predictive models.

## Introduction

Peripheral T-cell lymphoma (PTCL) represents a heterogeneous group of aggressive lymphomas arising from mature T-cells^1^. The incidence of PTCL is higher in Asia than in Europe, and risk factors such as genetic factors, immune abnormalities, environmental factors, and infectious causes have been proposed^2^. Epstein–Barr virus (EBV)-associated PTCL is more common in Asia, where EBV infection is associated with inferior outcomes^3^. CHOP (cyclophosphamide, doxorubicin, vincristine, prednisolone) or CHOP-like regimens such as THP-COP (pirarubicin, cyclophosphamide, vincristine, and prednisolone) are frequently used to treat patients with PTCL, but the prognosis has not been as good as that of patients with aggressive B-cell lymphoma^4^. In population-based studies, most PTCL patients progress or relapse following the first therapy, and long-term overall survival (OS) rates are within the range of 20–40%^5^. The more accurate the prognosis that can be determined during diagnosis or before treatment, the more appropriate the management that can be provided.

The International Prognostic Index (IPI) was developed in 1993 to identify patients with aggressive non-Hodgkin lymphoma (NHL) with poor prognosis^6^. Age, performance status (PS), lactate dehydrogenase (LDH), clinical stage, and extranodal involvement are used to calculate the IPI score. The IPI was primarily based on aggressive B-cell lymphoma rather than T-cell lymphoma but has shown an ability to stratify PTCL patients^7,8^. The Prognostic Index for PTCL-U (PIT) is a revised version of the IPI developed in 2004, mainly for PTCL, not otherwise specified (PTCL-NOS)^9^. The PIT includes three features of the IPI (age, PS, and LDH) along with bone marrow (BM) involvement. Although the PIT model was initially reported to offer better predictive ability than the IPI model, other studies found no difference between the two models^10–14^.

Since no studies have directly compared these two models using statistical methods, no conclusions have been reached regarding which model offers the better prognostic ability. Japan has the highest rate of aging in the world^15^, which brings with it many opportunities to treat elderly PTCL patients. However, relatively few studies have included high proportions of elderly PTCL patients. Therefore, we decided to directly compare the predictive powers of IPI and PIT in real-world Japanese patients with PTCL to determine which prognostic model is statistically superior.

## Results

### Patient characteristics

Of the 132 patients who met the inclusion criteria during the study period, we excluded 5 patients with missing clinical information and 14 who did not receive chemotherapy (Supplementary Fig. S1). For the remaining 113 patients, their characteristics at diagnosis are presented in Table 1. The median age was 67 years (range, 16–88 years), and 75 patients (66.4%) were male. The most common disease type was PTCL-NOS (69.0%), and the second most common was angioimmunoblastic T-cell lymphoma (AITL) (13.3%). Ninety patients (79.6%) had clinical stage III/IV, 29 (25.7%) had two or more extranodal lesions, and 27 (23.9%) showed BM involvement. Overall, 68 patients (60.2%) were categorized as high-intermediate or high risk by IPI, and 69 (61.1%) as group 3 or 4 by PIT. For most patients (95.6%), initial therapy comprised CHOP or THP-COP, and 12 patients (10.6%) received upfront autologous stem cell transplantation (auto-SCT).

**Table 1:** Patient characteristics at diagnosis.

	All patients (N = 113)
Age, years—median, range	67 (16–88)
Age > 60 years—n (%)	75 (66.4)
Male—n (%)	75 (66.4)
Diagnosis—n (%)	
PTCL-NOS	78 (69.0)
ALK-positive ALCL	9 (8.0)
ALK-negative ALCL	5 (4.4)
AITL	15 (13.3)
EATL	5 (4.4)
HSTL	1 (0.9)
ECOG PS ≥ 2—n (%)	33 (29.2)
B symptoms—n (%)	52 (46.0)
Elevated LDH (> ULN)—n (%)	71 (62.8)
Ann Arbor Stage III/IV—n (%)	90 (79.6)
Extranodal sites ≥ 2—n (%)	29 (25.7)
BM involvement—n (%)	27 (23.9)
IPI—n (%)	
Low (0, 1)	23 (20.4)
Low-intermediate (2)	22 (19.5)
High-intermediate (3)	42 (37.2)
High (4, 5)	26 (23.0)
PIT—n (%)	
Group 1 (0)	12 (10.6)
Group 2 (1)	32 (28.3)
Group 3 (2)	41 (36.3)
Group 4 (3, 4)	28 (24.8)
First treatment	
CHOP	59 (52.2)
THP-COP	49 (43.4)
Other	5 (4.4)
Upfront auto-SCT	12 (10.6)

*AITL* angioimmunoblastic T-cell lymphoma, *ALCL* anaplastic large cell lymphoma, *ALK* anaplastic lymphoma kinase, *BM* bone marrow, *EATL* enteropathy-associated T-cell lymphoma, *ECOG PS* Eastern Cooperative Oncology Group performance status, *HSTL* hepatosplenic T-cell lymphoma, *IPI* International Prognostic Index, *LDH* lactate dehydrogenase, *PIT* Prognostic Index for PTCL-U, *PTCL-NOS* peripheral T-cell lymphoma, not otherwise specified, *ULN* upper limit of normal.

### OS and progression-free survival (PFS)

**Figure 1:**
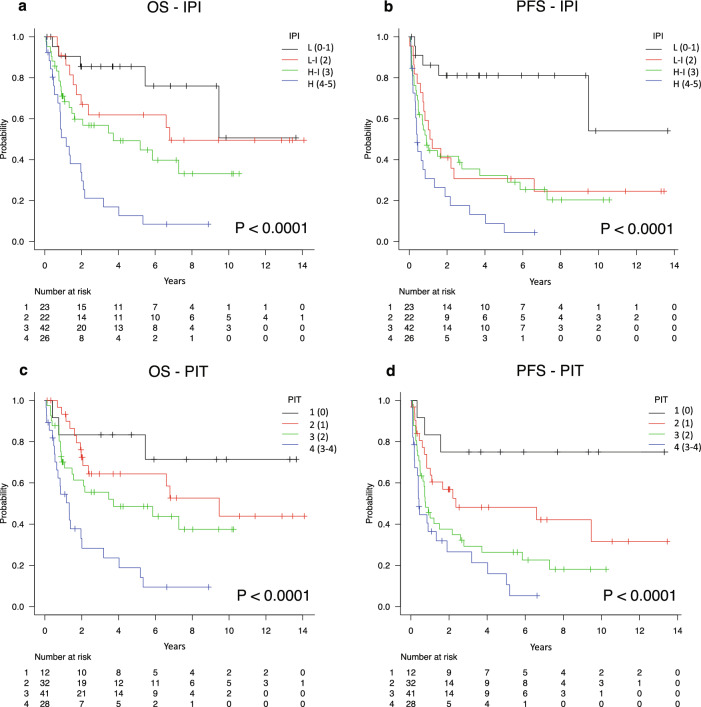
Kaplan–Meier curves of OS and PFS according to the IPI and PIT. (**a**,**b**) OS (**a**) and PFS (**b**) according to the IPI. (**c**,**d**) OS (**c**) and PFS (**d**) according to the PIT. *OS* overall survival, *PFS* progression-free survival, *IPI* International Prognostic Index, *PIT* Prognostic Index for PTCL-U.

During follow-up (median, 6.8 years; interquartile range, 2.7–9.9 years), 59 patients died. The cause of death was progression of lymphoma in 39 patients and other in 20 patients. Median OS was 4.0 years (95% confidence interval [CI] 2.0–7.3 years) and median PFS was 1.2 years (95%CI 0.7–2.6 years) (Supplementary Fig. S2). Five-year OS rates for the four IPI groups were 85.4%, 61.8%, 49.2%, and 12.7%, respectively (*P* < 0.0001), and for the four PIT groups were 83.3%, 64.4%, 48.6%, and 18.9%, respectively (*P* < 0.0001). Five-year PFS rates for the four IPI groups were 81.1%, 30.7%, 32.2%, and 8.8%, respectively (*P* < 0.0001), and similarly, for the four PIT groups were 75.0%, 48.1%, 26.4%, 16.0%, respectively (*P* < 0.0001) (Fig. 1).

### Comparison of prognostic impacts of IPI and PIT

**Figure 2:**
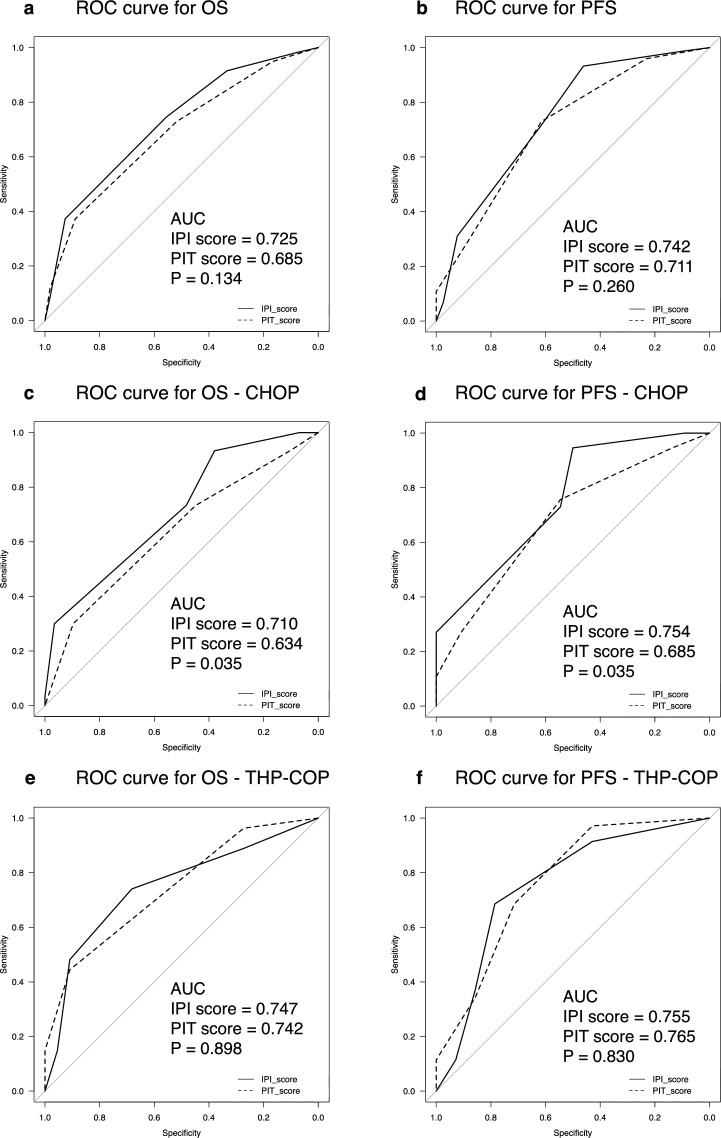
Comparison of ROC curves between the IPI and PIT. Solid lines represent the AUC for the IPI, and dotted lines represent the AUC for the PIT. (**a**,**b**) OS (**a**) and PFS (**b**) in all patients. (**c,d**) OS (**c**) and PFS (**d**) in patients treated with CHOP. (**e**,**f**) OS (**e**) and PFS (**f**) in patients treated with THP-COP. *OS* overall survival, *PFS* progression-free survival, *ROC* receiver operating characteristic, *AUC* area under the curve, *IPI* International Prognostic Index, *PIT* Prognostic Index for PTCL-U.

When receiver operating characteristic (ROC) curves for OS were generated, and areas under the curve (AUCs) were compared, AUC for IPI score was 0.725 (95%CI 0.636–0.814) and AUC for PIT score was 0.685 (95%CI 0.593–0.778), with no significant difference between scores (*P* = 0.134) (Fig. 2a). For PFS, the AUC for IPI score was 0.742 (95% CI 0.646–0.837), and the AUC for PIT score was 0.711 (95% CI 0.614–0.808), with no significant difference between scores (*P* = 0.260) (Fig. 2b). In a subgroup analysis of patients treated with CHOP, the AUC for IPI score was significantly higher than that for PIT score for both OS (0.710 vs. 0.634, *P* = 0.035) and PFS (0.754 vs. 0.685, *P* = 0.035) (Fig. 2c,d). On the other hand, among patients treated with THP-COP, no significant difference in AUC was detected between IPI and PIT scores for both OS (0.747 vs. 0.742, *P* = 0.898) and PFS (0.755 vs. 0.765, *P* = 0.830) (Fig. 2e,f).

**Figure 3:**
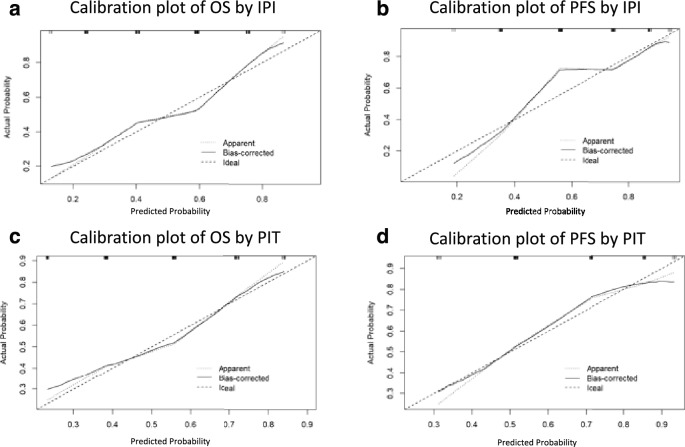
Calibration plot for OS and PFS by IPI and PIT. (**a**) OS according to the IPI. (**b**) PFS according to the IPI. (**c**) OS according to the PIT. (**d**) PFS according to the PIT. *OS* overall survival, *PFS* progression-free survival, *IPI* International Prognostic Index, *PIT* Prognostic Index for PTCL-U.

**Figure 4:**
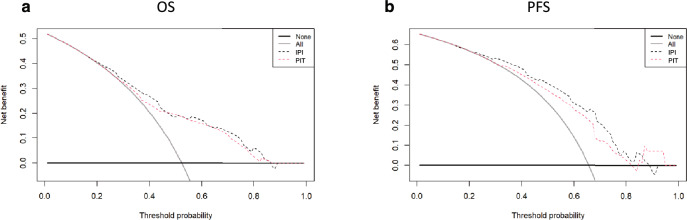
Decision curve analysis comparing the prognostic abilities of IPI and PIT in predicting (**a**) OS and (**b**) PFS. The gray line represents the net benefit of treating all patients, assuming all patients survive. The black line represents the net benefit if all patients were treated in the same way, assuming all patients die. The red dotted line represents the net benefit if patients were treated according to IPI. The black dotted line represents the net benefit if patients were treated according to PIT. *OS* overall survival, *PFS* progression-free survival, *IPI* International Prognostic Index, *PIT* Prognostic Index for PTCL-U.

In addition to the primary analyses, we conducted post-hoc evaluations using calibration plots and decision curve analyses to further compare the prognostic abilities of the IPI and PIT. Calibration plots for both OS and PFS showed no clinically relevant differences between the IPI and PIT (Fig. 3). Similarly, decision curve analyses for OS and PFS revealed nearly identical curves for both the IPI and PIT (Fig. 4).

### Multivariable analysis of IPI and PIT factors

Multivariable analysis of IPI and PIT factors using Cox proportional hazards modeling showed that Eastern Cooperative Oncology Group (ECOG) PS ≥ 2 (hazard ratio [HR] 3.30, 95% CI 1.82–6.01; *P* < 0.0001) and extranodal lesions ≥ 2 (HR 2.05; 95% CI 1.08–3.90; *P* = 0.029) were significantly associated with OS and ECOG PS ≥ 2 (HR 1.84, 95% CI 1.1.0–3.07; *P* = 0.021) was significantly associated with PFS. Clinical stage III/IV and BM involvement were not significantly associated with OS or PFS (Table 2). We performed multivariable analysis with B symptoms and upfront auto-SCT as covariates in a post-hoc analysis. The results showed that clinical stage III/IV was significantly associated with PFS (HR 2.49, 95% CI 1.08–5.79, *P* = 0.033), but other results were generally the same.

**Table 2:** Multivariable COX proportional hazards analyses of overall survival and progression-free survival.

Factor	Overall survival		Progression-free survival	
	HR (95%CI)	*P* value	HR (95%CI)	*P* value
Age > 60 years	1.70 (0.87–3.34)	0.12	1.74 (0.96–3.15)	0.067
ECOG PS 2–4	3.30 (1.82–6.01)	< 0.001	1.84 (1.10–3.07)	0.021
Elevated LDH (> ULN)	1.31 (0.71–2.39)	0.39	1.38 (0.80–2.36)	0.25
Extranodal sites ≥ 2	2.05 (1.08–3.90)	0.029	1.25 (0.70–2.24)	0.45
Stage III or IV	1.44 (0.57–3.63)	0.44	2.30 (1.00–5.29)	0.05
BM involvement	0.84 (0.44–1.62)	0.61	1.15 (0.65–2.05)	0.63

*ECOG PS* Eastern Cooperative Oncology Group performance status, *BM* bone marrow, *CI* confidence interval, *HR* hazard ratio, *LDH* lactate dehydrogenase, *ULN* upper limit of normal.

### Comparison of IPI and PIT with NCCN-IPI

We compared the National Comprehensive Cancer Network (NCCN)-IPI with IPI and PIT in a post-hoc analysis. NCCN-IPI was able to stratify PTCL patients into four groups for OS and PFS (Supplementary Fig. S3). Comparing the IPI and NCCN-IPI using ROC curves revealed no significant differences in AUCs for OS (0.725 vs. 0.721, *P* = 0.900) or PFS (0.742 vs. 0.732, *P* = 0.752) (Supplementary Fig. S4a,b). Similarly, when comparing PIT and NCCN-IPI using ROC curves, no significant differences in AUCs were found for OS (0.685 vs. 0.721, *P* = 0.187) or PFS (0.711 vs. 0.732, *P* = 0.732) (Supplementary Fig. S4c,d).

## Discussion

In the present study, we have demonstrated no significant differences in the predictive abilities of IPI and PIT for PTCL. We also showed that IPI and PIT offer applicable predictive models for PTCL in real-world practice with a large elderly population. Given the lack of difference between the IPI and PIT, using the PIT with its four indices (age, PS, LDH, and BM involvement) is easier than IPI using five indices (age, PS, LDH, extranodal involvement, and clinical stage). In terms of prognostication, BM biopsy has shown the potential to simplify testing (e.g., PET-CT and endoscopy) for staging and the assessment of extranodal disease. However, we cannot conclude that BM involvement alone is sufficient for evaluation, because multivariable analysis did not identify BM involvement as an independent prognostic factor, whereas the presence of two or more extranodal lesions was a significant prognostic factor.

In addition to age (> 60 years), ECOG PS (≥ 2), and LDH (above the upper limit of normal) identified by the IPI, PIT, which was proposed by the Intergruppo Italiano Linfomi, identified BM involvement as a poor prognostic factor^9^. In that study, the PIT model was described as offering superior predictive ability compared to the IPI based on the superior log-rank test statistic (66.79 vs. 55.94), but no direct statistical comparisons were made^9^. BM infiltration was considered a prognostic factor in the univariate analysis when the IPI was developed but was not selected when the prognostic model was created^6^. In addition, Weisenburger et al. retrospectively examined 340 PTCL-NOS patients and found that BM infiltration did not represent a robust predictor of OS (*P* = 0.03). Based on the results of the present analysis and previous reports^11^, PIT, which assesses BM infiltration, may not be clearly superior to IPI for predicting the survival of PTCL patients. Indeed, the presence of ≥ 2 extranodal lesions, but not BM infiltration, was significantly associated with OS in the present study. On the other hand, BM infiltration has been reported as a prognostic factor for patients with PTCL who undergo upfront auto-SCT^16^, indicating that its impact may vary depending on the treatment modality. In our study, only 12 patients (10.6%) underwent upfront auto-SCT, so BM infiltration may not have had a significant impact on prognosis. Prognostic factors for PTCL can differ depending on the treatment regimen. In a subgroup analysis focused on treatment regimens, the IPI demonstrated significantly greater accuracy in prognostic predictions than the PIT when treating PTCL with CHOP. Conversely, no significant difference between the IPI and PIT was observed for PTCL cases treated with THP-COP. As brentuximab vedotin combined with CHP (BV-CHP) is currently the standard of care for CD30-positive PTCL and anaplastic large-cell lymphoma (ALCL)^17^, the efficacy of the IPI and PIT within this treatment context warrants further investigation.

Various other prognostic factors for PTCL have been investigated. For instance, the International Peripheral T-Cell Lymphoma Project score (IPTCLP) was investigated for PTCL-NOS and AITL^18^. This prognostic index included age, PS, and platelet cell count (≤ 150 × 10^9^/L vs. > 150 × 10^9^/L) to divide patients into four groups. This study showed inferior OS in all but the low-risk group for the three indices of IPI, PIT, and IPTCLP. The modified PIT (m-PIT) is an updated version of the PIT for PTCL-NOS and AITL^19^. The m-PIT included age, PS, LDH, and Ki-67 (≤ 80% vs. > 80%) to divide patients into three groups. This score was associated with patient outcome (*P* < 0.0001) and was found to be more robust than PIT (*P* = 0.0043), but direct statistical comparisons to the PIT were not made. Gutiérrez-García et al. compared four prognostic indices (IPI, PIT, IPTCLP, m-PIT) in 122 PTCL patients (22 with ALCL, 56 with PTCL-NOS, 44 with others) ^10^. Multivariable analysis including these four prognostic indices identified IPTCLP as the most important prognostic factor for predicting OS (relative risk [RR] 3.52, 95%CI 2.01–7.12, *P* = 0.0001). In a similar analysis limited to 56 PTCL-NOS patients, IPTCLP was also the most significant prognostic factor (RR 7.69, 95%CI 2.21–13.17, *P* = 0.002). Yamasaki et al. performed a retrospective analysis comparing four prognostic indices (IPI, PIT, IPTCLP, m-PIT) in PTCL-NOS (n = 100) and AITL (n = 128) patients, revealing that better c-statistics (> 0.7) were only found for the IPI score for OS in PTCL-NOS^14^. However, in a study comparing three prognostic indices (IPI, PIT, and NCCN-IPI) in patients with ALCL without anaplastic large-cell lymphoma kinase (ALK) (n = 152), AITL (n = 145) and PTCL-NOS (n = 306), NCCN-IPI appeared to separate prognostic groups, although this difference did not result in markedly better c-statistics^13^. Therefore, even evaluating these reports overall, no conclusion has been reached regarding which prognostic index is best for patients with PTCL.

This study was conducted as a multicenter cohort study, which allowed us to collect many patients with PTCL and conduct long-term follow-up. However, the study had several limitations. First, this was a retrospective study and may have included selection bias. Second, this study included numerous disease types, so we could not study any specific disease type. Third, comparisons or combinations with other prognostic indicators, such as IPTCLP and m-PIT, were not examined. Additional validation using a larger collection of cases is needed in the future.

In conclusion, IPI and PIT are useful as predictive models for PTCL, with no models showing significant differences in prognostic performance.

## Methods

### Patient cohort

The Gifu Hematology Study Group in Japan retrospectively collected data for patients diagnosed with PTCL from six centers (Gifu University Hospital, Gifu Municipal Hospital, Gifu Prefectural General Medical Center, Gifu Red Cross Hospital, Takayama Red Cross Hospital, Gihoku Kosei Hospital) during the study period from June 2004 to December 2019. All PTCL cases that met the histologically definitive criteria of the 2016 revision of the World Health Organization classification^20^ and were at least 18 years old were included. PTCL-NOS, AITL, ALCL with or without ALK, enteropathy-associated T-cell lymphoma, and hepatosplenic T-cell lymphoma were included as PTCL subtypes. Patients with human immunodeficiency virus or diagnosed with adult T-cell leukemia/lymphoma or extranodal natural killer/T-cell nasal type were excluded. Patients who received only best supportive care, such as steroids, antibiotics, analgesics, antiemetics, blood transfusions, or palliative irradiation, were also excluded.

The examined variables included sex, age, ECOG PS, B symptoms (unexpected weight loss, fever, night sweats), serum LDH levels, Ann Arbor stage, number of extranodal sites, and BM involvement. Outcome variables included first chemotherapy, best response, use of upfront auto-SCT, and relapse or disease progression after treatment. Treatment was determined by the attending physician. This study was conducted in compliance with the Declaration of Helsinki. This study was reviewed by the Medical Review Board of Gifu University Graduate School of Medicine, which waived the need to obtain written informed consent based on the retrospective design of the study (approval number. 2019-255). The institutional review board at each participating site approved the protocol before data collection.

### IPI, PIT and NCCN-IPI

Age (≤ 60 years vs. > 60 years), ECOG PS (≤ 1 vs. > 2), LDH level (normal vs. above normal), Ann Arbor stage (I-II vs. III-IV), and extranodal involvement (≤ 1 vs. > 2) were the factors used to calculate IPI^6^. IPI identified four risk categories: low risk (score 0–1), low-intermediate risk (score 2), high-intermediate risk (score 3), and high risk (score 4–5). Age (≤ 60 years vs. > 60 years), ECOG PS (≤ 1 vs. > 2), LDH level (low vs. high), and BM involvement (negative vs. positive) were the factors used to calculate PIT^9^. Depending on the number of adverse prognostic factors, patients were classified into low-risk (0), low-intermediate risk (1), high-intermediate risk (2), or high-risk (≥ 3) groups. Age (41–60 years: 1; 61–75 years: 2; > 75 years: 3), ECOG PS (> 2: 1), LDH ratio to normal (> 1–3: 1, > 3: 2), Ann Arbor stage (III–IV: 1), and extranodal involvement (involvement BM, central nervous system, liver/gastrointestinal tract or lung: 1) were the factors used to calculate the NCCN-IPI^21^. Patients were classified according to the total NCCN-IPI score into low-risk (total score 0–1), low intermediate-risk (total score 2–3), high intermediate-risk (total score 4–5), or high-risk (total score 6–8) groups.

### Statistical analysis

The treatment outcomes were OS and PFS. OS was calculated from diagnosis to last follow-up or death from any cause. PFS was calculated from diagnosis to first occurrence of progression, relapse after response, or death from any reason. At the last observation, patients who were lost to follow-up were censored. Median follow-up was determined based on the reverse Kaplan–Meier method and was given as median and interquartile range. Continuous variables are presented as median and range, and the Mann–Whitney U test was used for group comparisons. Categorical variables are presented as number and percentage, and the chi-squared or Fisher’s exact test was performed for group comparisons, as appropriate. Survival curves were estimated using Kaplan–Meier methods and compared by log-rank testing. The prognostic abilities of IPI and PIT were compared using the AUC of the ROC curve^22^. Model calibration was assessed to evaluate the agreement between predicted probabilities generated by the IPI and PIT models and observed probabilities for both OS and PFS. The calibration plot was constructed by plotting predicted probabilities against observed probabilities. Decision curve analysis quantified the clinical net benefit of using the IPI and PIT prognostic models for predicting OS and PFS^23^. Multivariable Cox proportional hazards modeling was performed using IPI and PIT items. A two-sided *P* < 0.05 was considered significant. Statistical analyses were performed using EZR version 1.37^24^.

### Supplementary Information

Supplementary Information.

## Data Availability

The datasets generated during the current study are available from the corresponding author on reasonable request.
