# Supplementary material for: Comparison of the prognostic impact of IPI and PIT in peripheral T-cell lymphoma in real-world practice with a large elderly population
Source: Sci Rep. 2023 Nov 4;13:19060. doi: 10.1038/s41598-023-46501-5 (PMC10625631; doi:10.1038/s41598-023-46501-5)
Supplement: Supplementary file 1 — Supplementary Information. [file 41598_2023_46501_MOESM1_ESM.docx]

**Comparison of the Prognostic Impact of IPI and PIT in Peripheral T-cell Lymphoma in Real-World Practice with a Large Elderly Population**

Nobuhiko Nakamura^1*^, Nobuhiro Kanemura^1^, Takuro Matsumoto^1^, Hiroshi Nakamura^1^, Yoshikazu Ikoma^1,2^, Yuhei Shibata^2^, Junnichi Kitagawa^2^, Senji Kasahara^2^, Toshiki Yamada^3^, Michio Sawada^4^, Yuto Kaneda^1,5^, Kenji Fukuno^5^, Eri Takada^6^, Hideko Goto^7^, Shin Lee^8^, Kei Fujita^8^, Tetsuji Morishita^8^, Takeshi Hara^8^, Hisashi Tsurumi^1,8^ and Masahito Shimizu^1^

^1^ Department of Hematology and Infectious Disease, Gifu University Hospital, Gifu, Japan

^2^ Department of Hematology, Gifu Municipal Hospital, Gifu, Japan

^3^ Department of Hematology, Gifu Prefectural General Medical Center, Gifu, Japan

^4^ Department of Hematology, Gifu Red Cross Hospital, Gifu, Japan

^5^ Department of Hematology, Takayama Red Cross Hospital, Takayama, Japan

^6^ Department of Hematology, Gihoku Kosei Hospital, Yamagata, Japan

^7^ Department of Hematology, Chuno Kosei Hospital, Seki, Japan

^8^ Department of Hematology, Matsunami General Hospital, Gifu, Japan

* nakamura.nobuhiko.s1@f.gifu-u.ac.jp

**Supplemental information**

**Figure S1.** Patient flow. PTCL: peripheral T-cell lymphoma.

**Figure S2.** Kaplan–Meier curves for OS (a) and PFS (b). OS: overall survival; PFS: progression-free survival.

**Figure S3.** Kaplan–Meier curves for OS (a) and PFS (b) according to NCCN-IPI. OS: overall survival; PFS: progression-free survival; NCCN-IPI: National Comprehensive Cancer Network International Prognostic Index.

**Figure S4.** Comparison of ROC curves between IPI, PIT and NCCN-IPI. Solid lines represent the AUC for IPI or PIT, and dotted lines represents the AUC for NCCN-IPI. OS: overall survival; PFS: progression-free survival; ROC: receiver operating characteristic; AUC: area under the curve; IPI: International Prognostic Index; PIT: Prognostic Index for PTCL-U; NCCN-IPI: National Comprehensive Cancer Network International Prognostic Index.

| **Table S1.** Post-hoc multivariable COX proportional hazards analysis of overall and progression-free survival | | | | | |
| --- | --- | --- | --- | --- | --- |
|  | Overall survival | |  | Progression-free survival | |
| Factor | HR (95%CI) | *P* value |  | HR (95%CI) | *P* value |
| Age >60 years | 1.39 (0.70–2.75) | 0.34 |  | 1.46 (0.80–2.66) | 0.21 |
| ECOG PS 2–4 | 3.25 (1.75–6.06) | <0.001 |  | 1.72 (1.00–2.94) | 0.048 |
| B symptoms | 0.88 (0.45–1.72) | 0.71 |  | 0.96 (0.53–1.74) | 0.88 |
| Elevated LDH (>ULN) | 1.32 (0.71–2.43) | 0.38 |  | 1.42 (0.82–2.47) | 0.21 |
| Extranodal sites ≥2 | 2.40 (1.20–4.81) | 0.014 |  | 1.39 (0.75–2.58) | 0.29 |
| Stage III or IV | 1.60 (0.63–4.08) | 0.32 |  | 2.49 (1.08–5.79) | 0.033 |
| BM involvement | 0.81 (0.38–1.73) | 0.59 |  | 1.15 (0.58–2.28) | 0.68 |
| Upfront auto-SCT | 0.32 (0.09–1.17) | 0.085 |  | 0.40 (0.15–1.10) | 0.075 |
| ECOG PS, Eastern Cooperative Oncology Group performance status; BM, bone marrow; CI, confidence interval; HR, hazard ratio; LDH, lactate dehydrogenase; ULN, upper limit of normal; auto-SCT, autologous stem cell transplantation. | | | | | |
